# Erratum: Medical physics staffing for radiation oncology: a decade of experience in Ontario, Canada

**DOI:** 10.1120/jacmp.v13i2.3915

**Published:** 2012-03-08

**Authors:** Jerry J. Battista, Brenda G. Clark, Michael S. Patterson, Luc Beaulieu, Michael B. Sharpe, L. John Schreiner, Miller S. MacPherson, Jacob Van Dyk

**Affiliations:** ^1^ Medical Physics London Regional Cancer Program London ON; ^2^ Radiation Medicine Program The Ottawa Hospital Cancer Centre Ottawa ON; ^3^ Juravinski Cancer Centre and McMaster University Hamilton ON; ^4^ Université Laval Québec QC; ^5^ Radiation Medicine Program, Princess Margaret Hospital University of Toronto Toronto ON; ^6^ Cancer Centre of South Eastern Ontario Kingston ON Canada

This Appendix and a staffing spreadsheet were inadvertently omitted from Volume 13 Number 1. The spreadsheet is included as a Supplementary File.

## APPENDIX A: STAFF CATEGORIES

To avoid ambiguity and to facilitate the use of the Ontario staffing algorithm, we define each job category.

***Certified Medical Physicist (Radiation Oncology):*** A medical physicist with specialized training in the application of ionizing radiation to the treatment of human disease. This individual in Ontario normally possesses a PhD degree and has a minimum of two years clinical experience, preferably in a structured residency training program. Clinical certification is based on national standards and administered in Canada by the Canadian College of Physicists in Medicine (CCPM). Responsibilities include equipment commissioning and quality assurance, treatment planning, radiation protection, clinical development and training of future radiation specialists (physicists, radiation therapists, dosimetrists, and radiation oncologists).

***Medical Physics Assistant (or Associate or Technologist):*** This individual provides technical assistance directly to medical physicists and normally possesses a BSc or MSc degree in the physical sciences, with additional on‐the‐job‐training in radiation dosimetry, treatment planning, and radiation safety. The physics assistant often performs quality assurance tests and other measurements outside clinical hours of operation, with the data subsequently reported to a supervisory physicist for review.

***Medical Dosimetrist (or Treatment Planner):*** This individual specializes in computerized radiation treatment planning and normally possesses either a BSc degree in physics or a diploma or degree from a radiation therapy training program. Responsibilities include all aspects of production of patient‐specific dose distributions in compliance with protocols specified by radiation oncologists and medical physicists. The administrative reporting lines vary across the Ontario centers, often with dual reporting to physics on technical issues and also to radiation therapy for professional and human resource issues.

***Engineering Technologist:*** This individual provides technical services in maintaining, designing, and constructing the major equipment and devices used in radiation oncology. The technologist normally possesses a college diploma, certificate, or license in electronics or mechanical engineering technology. Responsibilities include response to accelerator breakdowns and on‐site repairs, interfacing with vendors of the equipment. In Ontario, most of the equipment servicing is currently done in‐house by local engineering support, with only partial support for parts and external consultation through vendor service contracts. These technologists usually receive additional technology‐specific training by vendors of radiation therapy‐related equipment. Historically, this hybrid model of technical support has been demonstrated to provide rapid response with minimal equipment downtime in a very cost‐effective manner for Ontario. Centers that rely entirely on vendor support may require fewer in‐house engineering technologists.

***Information Technology Specialist (Radiation Oncology)***: This individual provides information technology support directly to the radiation oncology team and normally possesses a BSc or MSc in computer science with specialized training in hardware and software development and maintenance. Equipment includes computerized treatment planning systems, record and verify systems (radiation oncology information system), and networks for the transfer, storage, and backup of large datasets used in radiation oncology. This individual is not responsible for general networking infrastructure and support of generic office applications (i.e., “desk‐top” functions) normally provided centrally by the hospital information group. However, a collaborative interface with the hospital group on equipment selection, installations, network configurations, and back‐up procedures is important to maintain continuity of service and compatibility.

## Supporting information

Supplementary Material

